# Selenium-Rich Yeast Peptide Fraction Ameliorates Imiquimod-Induced Psoriasis-like Dermatitis in Mice by Inhibiting Inflammation via MAPK and NF-κB Signaling Pathways

**DOI:** 10.3390/ijms23042112

**Published:** 2022-02-14

**Authors:** Hengke Guo, Min Li, Hongmei Liu

**Affiliations:** 1 Hubei Key Laboratory of Bioinorganic Chemistry and Materia Medica, School of Chemistry and Chemical Engineering, Huazhong University of Science and Technology, Wuhan 430074, China; guohengke@hust.edu.cn (H.G.); d201980129@hust.edu.cn (M.L.); 2 Hubei Engineering Research Center for Biomaterials and Medical Protective Materials, Wuhan 430074, China

**Keywords:** selenium-rich yeast peptide fraction, psoriasis, inflammation, MAPK, NF-κB

## Abstract

Psoriasis, a chronic and immune-mediated inflammatory disease, adversely affects patients’ lives. We previously prepared selenium-rich yeast peptide fraction (SeP) from selenium-rich yeast protein hydrolysate and found that SeP could effectively alleviate ultraviolet radiation-induced skin damage in mice and inhibited H_2_O_2_-induced cytotoxicity in cultured human epidermal keratinocyte (HaCaT) cells. This study aimed to investigate whether SeP had a protective effect on imiquimod (IMQ)-induced psoriasis-like dermatitis in mice and the underlying mechanisms. Results showed that SeP significantly ameliorated the severity of skin lesion in IMQ-induced psoriasis-like mouse model. Moreover, SeP treatment significantly attenuated the expression of key inflammatory cytokines, including interleukin (IL)-23, IL-17A, and IL-17F, in the dorsal skin of mice. Mechanistically, SeP application not only inhibited the activation of JNK and p38 MAPK, but also the translocation of NF-κB into the nucleus in the dorsal skin. Furthermore, SeP treatment inhibited the levels of inflammatory cytokines and the activation of MAPK and NF-κB signaling induced by lipopolysaccharide in HaCaT cells and macrophage cell line RAW264.7. Overall, our findings showed that SeP alleviated psoriasis-like skin inflammation by inhibiting MAPK and NF-κB signaling pathways, which suggested that SeP would have a potential therapeutic effect against psoriasis.

## 1. Introduction

Psoriasis, a chronic immune-mediated inflammatory skin disease, affects over 60 million people worldwide [1,2]. Because of the symptoms, such as pain, itching, and bleeding, psoriasis has a negative influence on life quality and mental health of patients. Psoriasis was recognized by World Health Organization (WHO) as a “chronic, non-communicable, painful, disfiguring, and disabling disease for which there is no cure” in 2014 [2]. In psoriatic skin, inflammatory cytokines, produced by activated leucocytes, alter growth and differentiation of resident skin cells, especially keratinocytes [3]. Clinical and genetic studies focusing on the immune systems have highlighted the pivotal roles of inflammatory cytokines, such as interleukin (IL)-17, IL-23, and tumor-necrosis factor-α (TNF-α), and two fundamentally different cell types, including epidermal keratinocytes and mononuclear leukocytes, in the pathogenesis of psoriasis [1,4,5]. Moreover, nuclear factor-κB (NF-κB) and mitogen-activated protein kinases (MAPK) are the important potential molecular pathways involved in the pathogenesis of psoriasis through upregulating the pro-inflammatory cytokines and chemokines expression [4]. Psoriasis cannot be cured currently, and new therapies based on its pathogenesis are needed.

Recently, various food protein-derived bioactive peptides or protein hydrolysates have gained a lot of interest in nutraceutical, pharmaceutical, and cosmeceutical industries due to their myriad bioactivities, including antioxidant, anti-aging, and anti-inflammatory activities [6,7]. Particularly, the bioactive peptides with excellent anti-inflammatory activity and safety have attracted more and more attention in treating chronic inflammation diseases [8]. It has been reported that they exhibit anti-inflammatory activity by inhibiting inflammatory mediator expression and/or by modulating NF-κB and MAPK activation [9]. For example, the bioactive peptide derived from *Pyropia yezoensis* exerted anti-inflammatory activity by downregulating the MAPK pathway in lipopolysaccharides (LPS)-stimulated macrophage cell line RAW264.7 [10].

As an essential trace element for mammalian species, Selenium (Se) acts as an integral part of selenoproteins that exerts multiple effects, ranging from antioxidant and anti-inflammatory abilities to the active thyroid hormone secretion [11]. Se incorporation into small organic compounds can be safe for the therapy of various disease [12]. A large quantity of observational studies from human have proved that Se status is inversely associated with the severity of psoriasis. In 1989, it was first reported that Se concentrations in whole blood and plasma of psoriasis patients were decreased [13], and this result was confirmed in subsequent studies [14,15]. Considering their excellent biological functions, it can be speculated that combining Se with food-derived bioactive peptides might result in a synergistic effect. Thus, our previous work prepared the Se-rich yeast peptide fraction with lower molecular weight of <1 kDa (SeP) from Se-rich yeast protein hydrolysate. We found that SeP could effectively protect the dorsal skin against ultraviolet B radiation-induced damage in mice and protect cultured human epidermal keratinocyte cell line HaCaT against H_2_O_2_-induced cytotoxicity by inhibiting the activation of p38 MAPK [16]. Based on these results, we speculated that SeP may have a role in improving psoriasis.

Therefore, the present work investigated the effect of the topical application of SeP on imiquimod (IMQ)-induced psoriasis-like dermatitis in mice, which is a widely used mouse model of psoriasis [17,18]. Furthermore, LPS-induced HaCaT cells and RAW264.7 cells were used as in vitro models to explore the underlying mechanisms.

## 2. Results

### 2.1. SeP Alleviated the IMQ-Induced Psoriasis-like Dermatitis in Mice

Se-supplement ability of SeP in mice was evaluated by measuring liver Se content. The liver Se content in IMQ+SeP group was 1.6 ± 0.0 µg/g, which was markedly higher than that of CON (1.0 ± 0.1 µg/g) and IMQ (1.1 ± 0.1 µg/g) group (*p* < 0.001). These data demonstrated that SeP was absorbed through the dorsal skin and topical application of SeP enhanced the Se retention in the body.

**Figure 1:**
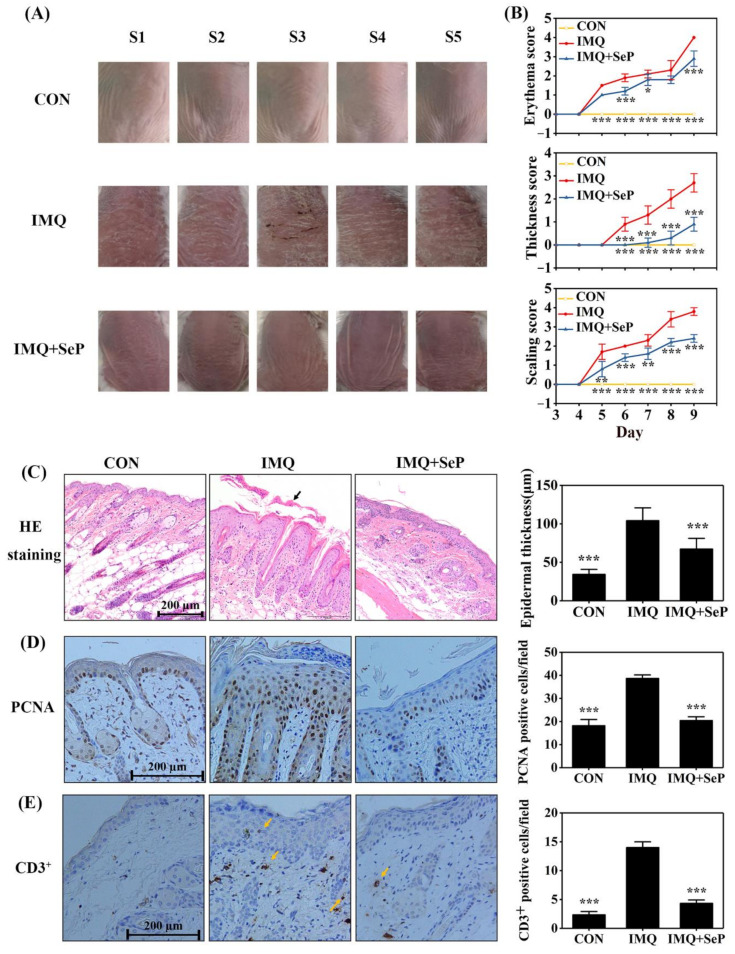
Effect of SeP on the IMQ-induced psoriasis-like dermatitis in mice. (**A**) The macroscopic appearance of mice back skin on day 9. The back skin of 5 mice (S1–5) were shown in each group. (**B**) The symptoms of erythema, skin thickness, and scaling were scored on day 3 to day 9 based on the PASI. (**C**) Hematoxylin-eosin (HE) staining of dorsal skin on day 9 (left panel) and quantification of the epidermal thickness (right panel). The black arrow indicated the sloughed scales. (**D**,**E**) Immunohistochemical staining for PCNA (**D**) and CD3 (**E**) in mouse dorsal skin on day 9. Representative images were shown in left panel and quantification of PCNA or CD3^+^ positive cells were shown in right panel. Quantification of the epidermal thickness, PCNA-positive cells and CD3^+^ positive cells of mouse back skin were obtained from 5 to 8 sites per mouse per group. Data were expressed as mean ± SD (*n* = 6). * *p* < 0.05, ** *p* < 0.01, *** *p* < 0.001, compared with the IMQ group.

The anti-psoriatic effect of SeP was evaluated by using IMQ-induced psoriasis-like dermatitis mouse model. As showed in Figure 1A, topical application of IMQ induced severe psoriatic phenotypes (erythema, scaling and thickening) on the dorsal skin of mice, which was alleviated by SeP treatment. In addition, the severity of lesions (erythema, skin thickness, and scaling) was evaluated according to psoriasis area and severity index (PASI). The PASI scores of erythema, skin thickness, and scaling were apparently increased in IMQ-induced mice from day 5 to day 9. However, SeP treatment elicited a reduction in PASI scores (Figure 1B).

In line with the macroscopic appearance changes, histopathological analysis of dorsal skin showed that the mice treated with IMQ had thicker epidermal layers than those of CON group (Figure 1C). Furthermore, immunohistochemical staining demonstrated that IMQ application induced epidermal hyperproliferation, as evidenced by the increased proliferating cell nuclear antigen (PCNA) positive cells (Figure 1D), and inflammatory cell infiltration, as evidenced by the increased CD3^+^ positive cells (Figure 1E). In contrast, compared with IMQ group, epidermal thickness, PCNA positive cells, and CD3^+^ positive cells were all markedly decreased in IMQ+SeP group (Figure 1C–E).

### 2.2. SeP Attenuated IMQ-Induced Systemic Side Effect in Mice

**Figure 2:**
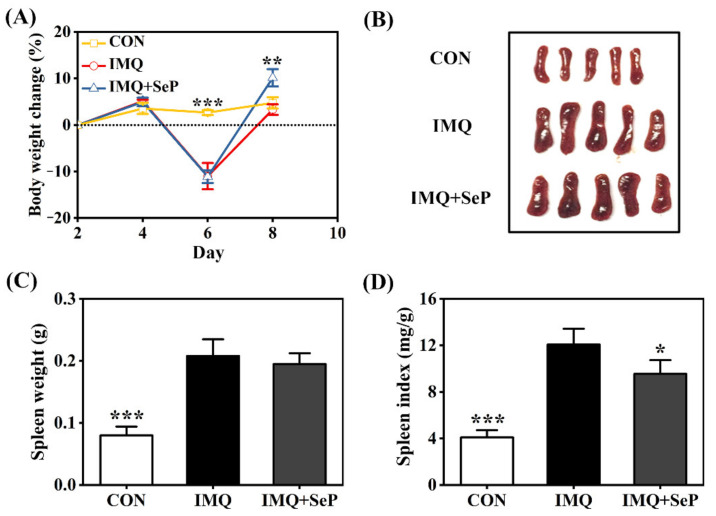
Influence of SeP on the IMQ-induced systemic side effects in mice. (**A**) Percent change of body weight of mice from day 2 to day 8. The body weight of mice on day 2 was used as baseline initial values in each group. (**B**) The photos of spleen tissues of each group (5 mice) on day 9. (**C**) Spleen weight. (**D**) Spleen index (spleen weight/body weight). Data were expressed as mean ± SD (*n* = 6). * *p* < 0.05, ** *p* < 0.01, *** *p* < 0.001, compared with the IMQ group.

The application of IMQ can lead to some systemic side effects like spleen enlargement and body weight loss in mice [19]. Consistently, on day 6, a remarkable body weight loss was observed in mice of IMQ and IMQ+SeP group after IMQ treatment for three days. However, an increase of body weight was observed in mice of IMQ+SeP group compared with that of IMQ group on day 8 (*p* < 0.01) (Figure 2A), indicating that SeP might have a beneficial effect on the health of the mice. At the end of the experiment, significant increases of spleen weight and index were found in mice of IMQ group compared with that of CON group (*p* < 0.001) (Figure 2B–D), but the spleen index in mice of IMQ+SeP group was significantly lower than that of IMQ group (Figure 2D).

### 2.3. SeP Inhibited the Expression of Psoriasis-Associated Molecules in the Dorsal Skin of IMQ-Induced Mice

**Figure 3:**
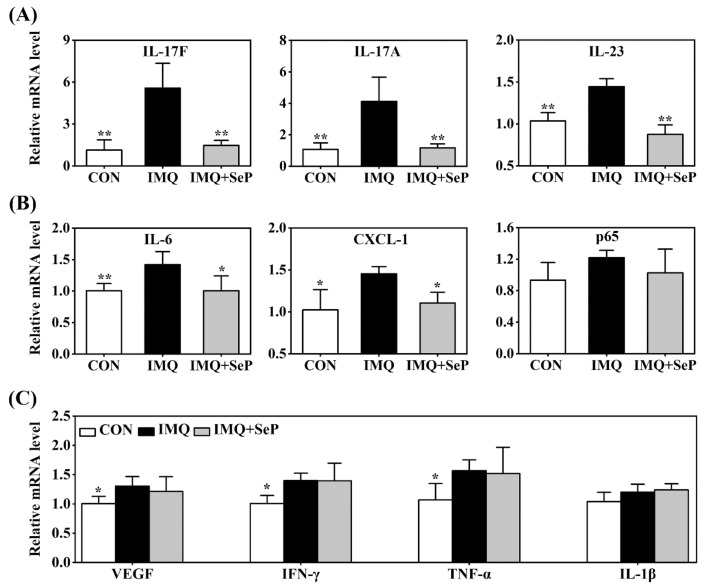
Effect of SeP on the expression of psoriasis-associated molecules in dorsal skin. The mRNA levels of IL-17F, IL-17A, IL-23 (**A**), IL-6, CXCL-1, p65 (**B**), VEGF, IFN-γ, TNF-α and IL-1β (**C**) were detected by RT-qPCR. Glyceraldehyde-3-phosphate dehydrogenase (GAPDH) was served as an internal reference. Data were expressed as the mean ± SD (*n* = 6). * *p* < 0.05, ** *p* < 0.01, compared with the IMQ group.

Many immune-derived cytokines and chemokines, including IL-17, IL-23, IL-6, C-X-C motif ligand-1 (CXCL-1), TNF-α, and p65, can drive inflammatory cell infiltration and an inflammation loop, thus regulating keratinocyte proliferation and leading to the development and maintenance of psoriasis [4]. Real-time quantitative polymerase chain reaction (RT-qPCR) was used to detect the expression levels of IL-17A, IL-17F, IL-23, IL-6, CXCL-1, p65, vascular endothelial growth factor (VEGF), interferon γ (IFN-γ), TNF-α, and IL-1β in the dorsal skin of mice. IMQ significantly upregulated mRNA levels of IL-17A, IL-17F, IL-23, IL-6, CXCL-1, VEGF, IFN-γ, and TNF-α (Figure 3A–C). SeP treatment significantly attenuated IMQ-induced upregulation of the IL-23/IL-17 axis related pro-inflammatory cytokines, including IL-17A, IL-17F, and IL-23 (*p* < 0.05 or *p* < 0.01), in mice skin (Figure 3A). In line with these results, SeP treatment displayed a blockade on the expression of IL-6 and CXCL-1 (Figure 3B). These results revealed that SeP treatment had a preventive effect on IMQ-induced skin inflammation.

### 2.4. SeP Suppressed the Activation of MAPK and NF-κB Pathways in the Dorsal Skin of IMQ-Induced Mice

**Figure 4:**
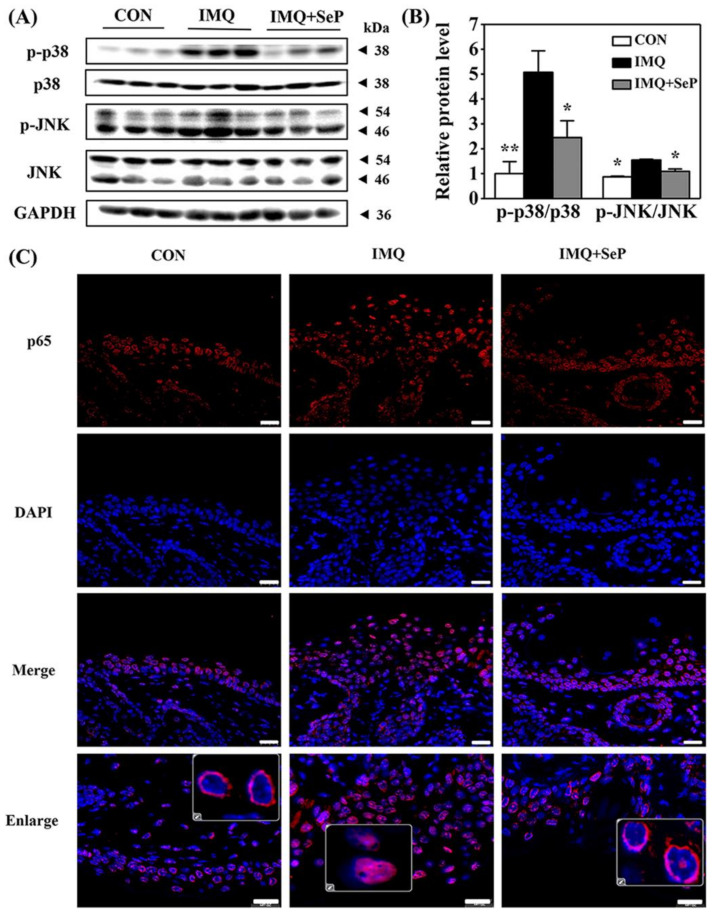
Effect of SeP on the activation of MAPK and NF-κB pathways in dorsal skin of IMQ-treated mice. (**A**,**B**) SeP inhibited the activation of MAPK induced by IMQ in the dorsal skin of mice. Western blot analysis was performed to measure the protein levels of p-p38, p38, p-JNK and JNK. (**A**) Representative image of bands. (**B**) Semi-quantification analysis of p-p38 normalized to p38 and p-JNK normalized to JNK (mean ± SD, *n* = 3). * *p* < 0.05, ** *p* < 0.01, compared with the IMQ group. (**C**) SeP inhibited the p65 translocation into the nucleus induced by IMQ in the dorsal skin of mice. Representative images of immunofluorescent staining of NF-κB p65. NF-κB p65 was stained in red with Cy3 and nucleus was stained blue with DAPI. Enlarge images showed the localization of p65. Scale bar = 20 µm.

To elucidate the mechanism by which SeP regulated the expression of cytokines and chemokines induced by IMQ, the protein levels of phosphorylated p38 MAPK (p-p38), p38, phosphorylated Jun N-terminal kinase (p-JNK), and JNK in the dorsal skin of mice were detected by Western blot assay. As shown in Figure 4A, IMQ application significantly increased the p-JNK and p-p38 expression levels, implying both p38 and JNK MAPK signaling activation. In contrast, the activation of p38 and JNK was strongly reversed by SeP treatment (Figure 4B).

Previous studies suggested that NF-κB plays a pivotal role in inflammatory and immune responses [20]. In resting cells, the inactive NF-κB is retained in the cytoplasm in complexes with the inhibitor protein IκB. Upon stimulation, IκB is phosphorylated and subsequently degraded by the proteasome, resulting in NF-κB translocation into the nucleus where they regulate the transcription of genes encoding pro-inflammatory cytokines [20]. Immunofluorescence analysis was performed to observe NF-κB p65 localization in dorsal skin of mice. As shown in Figure 4C, in the skin sections of mice from IMQ group, the fluorescent intensity of p65 was highly upregulated and p65 mainly localized in the nucleus. However, p65 mainly localized in the cytoplasm in the skin sections of mice from IMQ+SeP group, demonstrating that topical application of SeP inhibited the translocation of p65 into the nucleus.

### 2.5. SeP Inhibited LPS-Induced Pro-Inflammatory Cytokines Expression and Activation of MAPK and NF-κB Pathways in HaCaT Cells

It has been reported that hyperproliferative keratinocytes and inflammatory cytokines are involved in the complex etiology of psoriasis [4,21]. Moreover, LPS is believed to have the ability to induce the activation of MAPK and NF-κB, leading to the production of some pro-inflammatory cytokines, including TNF-α, IL-6, and IL-17, which may form an inflammatory microenvironment [22,23]. In order to further investigate the underlying mechanism of SeP against the psoriasis-like inflammation, LPS-stimulated HaCaT cells were used as in vitro model in present study.

**Figure 5:**
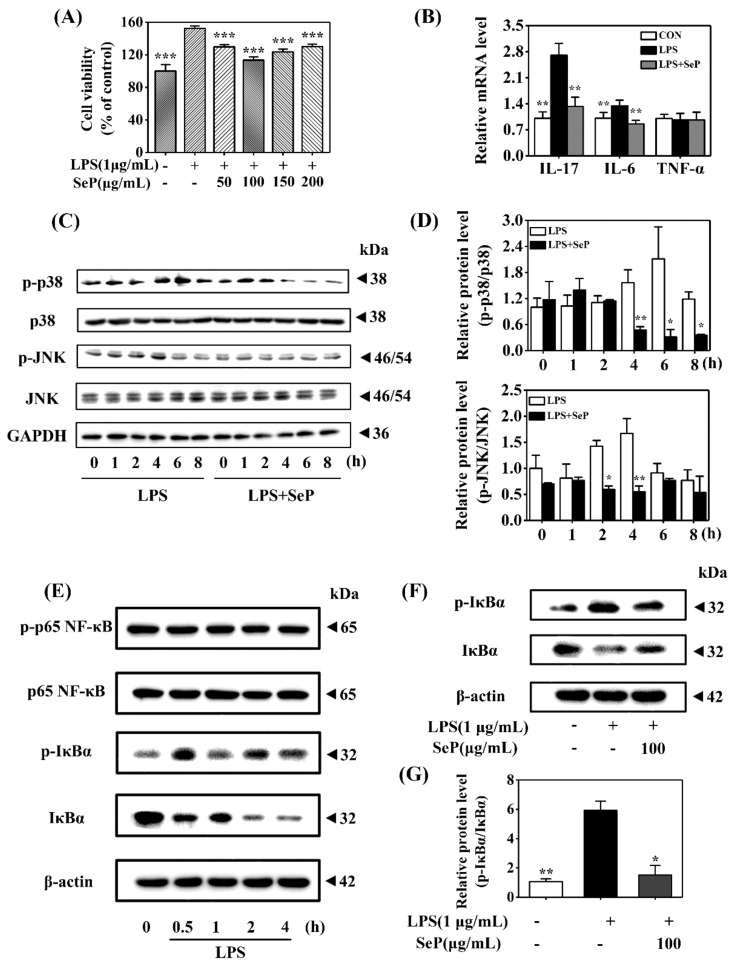
Effect of SeP on cell viability, pro-inflammatory cytokines expression, and the activation of MAPK and NF-κB signaling pathways in LPS-stimulated HaCaT cells. (**A**) SeP pretreatment suppressed the cell proliferation induced by LPS. After pretreated with indicated dose of SeP for 24 h, HaCaT cells were exposed to LPS (1 µg/mL) for another 24 h. MTT assay was used to determine the cell viability (means ± SD, *n* = 4). (**B**) SeP reduced the expression of IL-17A and IL-6 induced by LPS. Cells were pretreated with SeP (100 µg/mL) for 24 h and then exposed to LPS (1 µg/mL) for another 12 h. RT-qPCR assay was performed to measure the mRNA level of pro-inflammatory cytokines (means ± SD, *n* = 4). (**C**,**D**) SeP suppressed LPS-induced the activation of p38 and JNK MAPK in HaCaT cells. After pretreatment with or without SeP (100 µg/mL) for 12 h, cells were stimulated with LPS for another indicated times (0, 1, 2, 4, 6 and 8 h). Western blot analysis was used to determine the protein levels of p-p38, p38, p-JNK and JNK. (**C**) Representative Western blot bands of p-p38, p38, p-JNK and JNK. (**D**) Semi-quantification analysis of p-p38 MAPK normalized to p38 MAPK (upper panel) and p-JNK normalized to JNK (lower panel) (mean ± SD, *n* = 3). (**E**) LPS activated NF-κB signaling pathway in HaCaT cells. Cells were stimulated by LPS (1 µg/mL) for indicated times (0, 0.5, 1, 2 and 4 h), and the protein levels of phosphorylated p65 (p-p65), p65, p-IκBα and IκBα were detected by Western blot analysis. Representative Western blot bands were shown. (**F**,**G**) SeP suppressed LPS-induced the activation of NF-κB signaling pathway in HaCaT cells. Cells were pretreated with or without SeP (100 µg/mL) for 12 h and then stimulated with LPS (1 µg/mL) for another 0.5 h. Western blot analysis was done to measure the protein levels of p-IκBα and IκBα. (**F**) Representative Western blot bands. (**G**) Semi-quantification analysis of p-IκBα normalized to IκBα (mean ± SD, *n* = 3). Each band image was representative of three experiments. * *p* < 0.05, ** *p* < 0.01, *** *p* < 0.001, compared with cells treated with LPS only.

HaCaT cells underwent abnormal proliferation after exposure to LPS (1 µg/mL) for 24 h, as demonstrated by the increasing cell viability determined by MTT assay. SeP pretreatment in the range of 50–200 µg/mL markedly inhibited LPS-induced abnormal proliferation in HaCaT cells. The most inhibitory effect was achieved when the cells were pretreated with 100 µg/mL SeP for 24 h before exposure to LPS (Figure 5A). As shown in Figure 5B, compared with cells treated with LPS only, SeP pretreatment significantly suppressed the LPS-induced upregulation of IL-17A and IL-6 levels in HaCaT cells. No significant change of TNF-α expression was observed among all groups.

Western blot assay results revealed that LPS induced an increase of p-p38 level in HaCaT cells with maximal induction at 6 h. Similarly, LPS treatment also enhanced the level of p-JNK with maximal induction at 4 h. However, SeP pretreatment markedly decreased the levels of p-p38 and p-JNK induced by LPS and shortened the duration of p38 and JNK phosphorylation (Figure 5C,D). Moreover, LPS stimulated rapid phosphorylation of IκBα accompanied by its degradation but had a negligible effect on p65 phosphorylation (Figure 5E), suggesting that LPS activated the classical pathway of NF-κB signaling in HaCaT cells. SeP pretreatment significantly downregulated the activation of NF-κB induced by LPS, as evidenced by the decrease of IκBα phosphorylation and its degradation (Figure 5F,G).

Overall, these results demonstrated that SeP inhibited cell proliferation, pro-inflammatory cytokines expression, and activation of MAPK and NF-κB pathways induced by LPS in HaCaT cells.

### 2.6. SeP Reduced TNF-α Expression and p38 MAPK Activation Induced by LPS in RAW264.7 Cells

**Figure 6:**
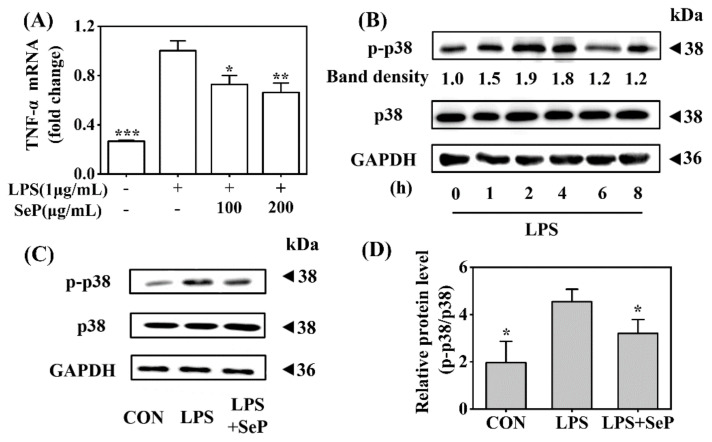
Effect of SeP on TNF-α expression and the activation of p38 MAPK signaling pathway in LPS-stimulated RAW264.7 cells. (**A**) SeP decreased LPS-induced TNF-α expression. After pretreated with SeP (100 and 200 µg/mL) for 4 h, HaCaT cells were exposed to LPS (1 µg/mL) for another 20 h. The mRNA expression level of TNF-α was measured by using RT-qPCR (means ± SD, *n* = 3). (**B**) LPS activated p38 MAPK signaling pathway. Cells were stimulated by LPS (1 µg/mL) for indicated times (0, 1, 2, 4, 6 and 8 h) and then the protein levels of p-p38 and p38 were measured by Western blot. Representative Western blot bands were shown. (**C**,**D**) SeP suppressed LPS-induced activation of p38 MAPK. Cells were pretreated with or without SeP (100 µg/mL) for 10 h and then stimulated with LPS (1 µg/mL) for another 2 h. Western blot analysis were performed to determine protein levels of p-p38 and p38. (**C**) Representative Western blot bands. (**D**) Semi-quantification analysis of p-p38 normalized to p38 (mean ± SD, *n* = 3). Each band image was representative of three experiments. * *p* < 0.05, ** *p* < 0.01, *** *p* < 0.001, compared with cells treated with LPS only.

Macrophages play a critical role in the maintenance of skin inflammation in psoriasis by the effective recruitment and activation of macrophages with sufficient release of TNF-α [24]. In this study, the anti-inflammatory effect of SeP was further investigated in LPS-stimulated RAW264.7 cells. As shown in Figure 6A, LPS induced a marked increase of TNF-α expression in RAW264.7 cells, which was significantly suppressed by SeP pretreatment at dose of 100 and 200 µg/mL. Western blot analysis results revealed that LPS induced the phosphorylation of p38 MAPK with maximal induction at 2 h (Figure 6B), which was significantly attenuated by SeP pretreatment (Figure 6C,D).

## 3. Discussion

In the present study, the protective effect of SeP on psoriasis-like skin inflammation was evaluated using IMQ-induced mouse model and LPS-induced in vitro cell culture models. We found that SeP application might suppress the expression of Th17 cell-associated pro-inflammatory cytokines through the inactivation of NF-κB and MAPK pathways, thus improving the psoriasis-like skin inflammation. These results suggested that SeP as a natural bioactive product with low toxicity might be a potential candidate for psoriasis prevention.

IMQ treatment in the back skin of mice can induce similar phenotypic and histological symptoms with human psoriasis lesions [17,18]. Thus, in this work, the effect of SeP on psoriasis-like skin inflammation was evaluated using this in vivo model. Studies have shown that SeP application significantly ameliorated the severity of skin lesion in IMQ-induced psoriasis-like mouse model. Firstly, SeP application ameliorated the macroscopic appearance change of dorsal skin, including erythema, skin thickness, and scaling (Figure 1A,B). Secondly, histopathological analysis of dorsal skin showed that SeP application inhibited IMQ-induced thickening of the epidermal layer (Figure 1C). Finally, immunohistochemical staining of dorsal skin demonstrated SeP application inhibited the increase of PCNA expression and CD3^+^ positive cell number induced by IMQ (Figure 1D,E), suggesting that SeP application reduced epidermal hyperproliferation and inflammatory cell infiltration in the lesional skin [25,26]. Additionally, the abnormal proliferation and differentiation of keratinocytes play a key role in the pathogenesis of psoriasis. The data from our in vitro experiments showed that SeP inhibited LPS-induced abnormal proliferation in human epidermal keratinocyte cell line HaCaT (Figure 5A). Taken together, all these results indicated that SeP might have a potent anti-psoriatic effect.

The anti-psoriatic effect of SeP might be partly attributed to its anti-inflammatory activity via decreasing the expression of psoriasis-related cytokine. The activation of IL23/Th17 cell inflammatory axis is a key event for the formation and development of psoriatic lesions [3,18,27,28]. IL-23, secreted by macrophages and dendritic cells, promotes the polar Th17 cell response and the subsequent release of pro-inflammatory cytokines, such as IL-17A, IL-17F, and TNF-α. IL-17, alone or synergistically with TNF-α acting on keratinocytes, results in the transcription of many psoriasis-related genes, including CCL20, CXCL1, -2, -3, and -8 chemokines [29]. Among them, CXCL-1 can induce the migration of neutrophils into the epidermis, thus propagating the psoriatic inflammation [30]. IL-6 plays a central role by cross-talk with cytokines of the IL-23/Th17 axis in the development of psoriasis [31]. Keratinocyte-derived cytokines such as VEGF influence the growth of supporting stromal cells, thus inducing the proliferation of keratinocytes [32]. IFN-γ in particular inhibits the apoptosis of keratinocytes, resulting in their hyper-proliferation in psoriatic skin [28]. In the present study, topically SeP application downregulated IMQ-induced overexpression of many pro-inflammatory factors including IL-17F, IL-17A, IL-23, IL-6 and CXCL-1 in dorsal skin of mice. Furthermore, the results from in vitro cell culture showed that SeP not only decreased LPS-induced IL-17A and IL-6 expression in HaCaT cells, but also decreased LPS-induced TNF-α expression in RAW264.7 cells. These results demonstrated that SeP ameliorated IMQ-induced psoriasis-like dermatitis probably by improving inflammation status. However, the limitation of this work is that the exact action mode of SeP is not clearly elucidated. A deeper study is needed to find the target of SeP in the future.

The anti-inflammatory activity of SeP might be achieved by inhibiting the activation of MAPK and NF-κB signaling pathways. Previous studies suggested that the increased MAPK and NF-κB activation promote epidermal hyperproliferation and exacerbate psoriatic pathogenesis by triggering inflammatory responses [4,33,34]. Particularly, p38 MAPK over-phosphorylation breaks down the homeostasis of skin and induces cellular stresses, which might be the trigger of psoriasis dermatitis [35,36]. NF-κB, indicated by the p65 subunit nuclear translocation and phosphorylation as well as the IκBζ ablation, is the key component of IL-17 mediated skin inflammation [37,38]. In this study, IMQ application not only increased the phosphorylation of p38 and JNK, but also promoted the translocation of p65 into the nucleus in dorsal skin of mice, suggesting the activation of MAPK and NF-κB signaling pathways. Excitingly, these effects of IMQ were significantly reversed by SeP treatment. Similarly, SeP reduced the phosphorylation of p38 and JNK and the degradation of IκBα in LPS-stimulated HaCaT cells, and the phosphorylation of p38 in LPS-stimulated RAW264.7 cells, further confirming the inhibitory effect of SeP on activation of MAPK and NF-κB. Therefore, SeP might improve inflammation status by inhibiting activation of MAPK and NF-κB, thereby alleviating IMQ-induced psoriasis-like dermatitis.

The anti-inflammatory activity of SeP might be contributed to its chemical composition. Our previous work shows that SeP prepared from Se-rich yeast protein hydrolysate contains both Se and bioactive peptides [16]. The contents of Leu and Glu in SeP are highest and SeMet is the main Se form in SeP (the content is more than 90% of total Se content). Moreover, the sequences of three peptides abundant in SeP, VLPVPF (Val-Leu-Pro-Val-Pro-Phe), LLPF (Leu-Leu-Pro-Phe), and FFPM (Phe-Phe-Pro-Met), were identified by liquid chromatography-tandem mass spectrometry (Table S1, Supplementary Materials). As a commonly used Se supplement, SeMet has anti-inflammatory effect in vivo and in vitro [11,39,40]. When women were given 200 μg per day SeMet, thyroid inflammatory activity fell, and post-partum thyroid disease and permanent hypothyroidism were significantly reduced [41]. In in vitro cultured U937 human macrophage cells, SeMet markedly suppressed the LPS-induced NF-κB activation, thereby exerting an anti-inflammatory activity [40]. On the other hand, food protein-derived bioactive peptides have been reported to exhibit anti-inflammatory activity by inhibiting the expression of inflammatory mediators and/or by modulating the activation of NF-κB and MAPK [9]. Importantly, several studies have showed that dipeptide pyroglutamyl-leucine (pyroGlu-Leu) could counteract the inflammatory response [42,43,44]. In an animal model, pyroGlu-Leu improved dextran sulfate sodium-induced colitis [42]. The results from in vitro cultured RAW 264.7 cells showed that pyroGlu-Leu inhibited the secretion of inflammatory mediators by suppressing IκBα degradation and MAPK (JNK, p38, and ERK) phosphorylation in stimulated by LPS [43]. Therefore, we speculated that SeMet and bioactive peptides (in particular Glu and Leu) might contribute to the anti-inflammatory activity of SeP. However, whether the anti-inflammatory activity of SeP is attributed to bioactive peptides or SeMet or the combination of the two needs to be clarified. Moreover, SeP is a mixture of peptides. Future research is needed to clarify what kind, or several kinds of peptide have anti-inflammatory activity.

Another limitation of this work is not to clarify SeP’s penetration through the skin barrier. The stratum corneum of the skin is the main barrier for percutaneous absorption of drug molecules. There are three possible routes available for molecules to pass the stratum corneum [45]. One is the transcellular pathway, requiring that molecules diffuse alternately through corneocytes and their lipid matrix. The second is the intercellular route, requiring that molecules cross the stratum corneum through various matrix environments. The third is an alternative route offered by sweat glands and hair follicles. Whatever the pathway, the penetration rate of a molecule through the stratum corneum barrier is strongly related to its physicochemical properties. Once the molecule has crossed the stratum corneum, it partitions into the dermis and diffuses through dermal tissue to reach the blood capillaries. Most molecules will pass the epidermal barrier through the intercellular route. As a consequence of its hydrophobic nature, the stratum corneum barrier will allow the penetration of lipid-soluble molecules more readily than water-soluble ones. However, strong lipophilic molecules will be impeded by the hydrophilic regions in the corneal layer. Water-soluble molecules may penetrate through an alternative route, the openings of sweat glands and hair follicles. The hydrophobic and hydrophilic performance of SeP is moderate based on its amino acid composition (rich in Leu, Gln, Asp, and Val) [16]. Moreover, the main molecular weight of SeP ranges from 200 to 550 Da which is in accord with the ‘500 Dalton rule’ for the skin penetration of molecules [46]. Therefore, it is speculated that SeP might penetrate the skin through the intercellular route or an alternative route.

## 4. Materials and Methods

### 4.1. Chemicals

IMQ cream (5%, Aldara) was purchased from Med-Shine Pharmaceutical Co., Ltd. (Sichuan, China). Bovine serum albumin (BSA) was purchased from Biosharp (Hefei, China). RIPA lysis buffer, phenylmethanesulfonylfluoride (PMSF) and antibodies against GAPDH, and β-actin were purchased from Beyotime (Jiangshu, China). LPS and 3-(4,5-dimethylthiazol-2-yl)-2,5-diphenyltetrazolium bromide (MTT) were obtained from Sigma-Aldrich (St. Louis, MO, USA). Trizol, Dulbecco’s Modified Eagle Medium (DMEM), RevertAid First Strand cDNA Synthesis Kit, and SYBR Green PCR Master Mix kit were purchased from Thermo Fisher Scientific (Waltham, MA, USA). The enhanced chemiluminescence (ECL) kit and polyvinylidene fluoride (PVDF) membrane were purchased from Millipore (Billerica, MA, USA). Fetal bovine serum (FBS) was purchased from Tianhang Biotechnology Co., Ltd. (Huzhou, China). Antibodies against p38 MAPK, p-p38, JNK, and p-JNK were purchased from Abcam (Cambridge, UK). Antibodies against p-p65, p65, phosphorylated IκBα (p-IκBα), and IκBα were purchased from Wanleibio (Shenyang, China). All chemicals were of analytical grade and used without further purification.

### 4.2. Preparation of SeP from Se-Rich Yeast Protein Hydrolysate

SeP was extracted from the Se-rich yeast protein hydrolysate as described in our previous work [16]. Se-rich brewer’s yeast (1900 μg Se/g) was kindly provided by Anhui Huaxin Biological Pharmaceutical Co., Ltd. (Jieshou, China).

### 4.3. In Vivo Animal Experiments

#### 4.3.1. Animals and Experimental Design

A total of 18 female BALB/c mice (age 6 weeks, weight 18 ± 2 g) were purchased from China Three Gorges University Research Center for Laboratory Animals (Yichang, Hubei, China). All animals were housed at room temperature (22–25 °C) under a 12:12 h light: dark cycle. The mice were fed water and food ad libitum. The animal operating protocols were followed in line with the institutional guidelines as well as the guidelines of the Animal Welfare Act and approved by the Institutional Animal Care and Use Committee at Huazhong University of Science and Technology. All efforts were made to minimize suffering.

After one week of adaptation to the laboratory conditions, mice were shaved on the dorsal skin over an area of 2 cm × 3 cm and randomly divided into three groups (*n* = 6) as follows: control group (CON), model group (IMQ) and experimental group (IMQ+SeP). IMQ cream (62.5 mg) was topically applied on the back skin of the mice daily for 6 consecutive days to develop a psoriasis-like dermatitis model, and the CON group mice were treated with the same amounts of Vaseline at the same intervals. The mice in IMQ+SeP group were applied topically with SeP solution (the peptide dose is 150 mg/kg body weight/day and the Se dose is 100 µg/kg body weight/day) on the dorsal skin 2 days before IMQ treatment until the sixth day of IMQ model. The mice in CON group and IMQ group received the equal volume of vehicle solution (distilled water) on the dorsal skin in line with the SeP treatment. The time interval between IMQ treatment and SeP treatment was 4–5 h daily. Mouse body weight was recorded every two days. The dorsal skin inflammation severity of mice was evaluated from the third day according to the method of PASI. Erythema, scaling, and thickness were scored independently on a scale ranging from 0 to 4 according to the degree of the inflammation: 0, none; 1, slight; 2, moderate; 3, marked; 4, very marked. The animal experiment schematic diagram is shown in Scheme S1.

At the end of the experiment, all mice were euthanized by pentobarbital sodium, and dorsal skin, spleen, liver, and whole blood were collected. Serum was separated from whole blood by centrifugation and stored in aliquots at −80 °C till further analysis. The dorsal skin, spleen and liver samples were isolated and washed twice with ice cold saline. The small sections of dorsal skin were fixed in 4% paraformaldehyde for histology, immunohistochemistry (IHC), and immunofluorescence analysis, and the remaining skin samples were stored at −80 °C for RNA extraction and Western blot assay. The liver samples were stored at −80 °C for Se concentration analysis.

#### 4.3.2. Histology, IHC and Immunofluorescence Analysis

The 4% paraformaldehyde-fixed dorsal skin was embedded in paraffin and then sectioned at a 3–4 µm thickness. Histological sections were stained with HE according to standard protocols, and the histopathological changes were observed and photographed under a light microscope (Olympus, Japan). The epidermal thickness was analyzed and calculated by Image-Pro Plus software (National Institutes of Health, Bethesda, MD, USA).

For IHC analysis of dorsal skin, the de-paraffinized sections were sequentially stained with primary antibody against CD3 or PCNA antibody and then horseradish peroxidase conjugated secondary antibody. The brown-yellow particles observed in the dermis or epidermis layer under a light microscope were considered as positive staining. The positive staining area was analyzed and calculated by Image-Pro Plus software.

For immunofluorescence analysis of dorsal skin, the de-paraffinized sections were stained with primary antibody against p65, and then incubated with Cy3 conjugated secondary antibody (goat anti-rabbit antibody at a 1:300 dilution). The DAPI was used to stain the nucleus. The staining results were observed under the fluorescence microscope (Nikon eclipse C1, Tokyo, Japan). All reagents used in immunohistochemistry and immunofluorescence analysis were purchased from Wuhan Servicebio Technology Co., Ltd. (Wuhan, China).

#### 4.3.3. Determination of Liver Se Content

The Se content in liver was determined by fluorescence method [47].

#### 4.3.4. Determination of Gene Expression by RT-qPCR

The mRNA levels of IL-17A, IL-17F, IL-23, IL-6, IL-1β, TNF-α, CXCL-1, IFN-γ, VEGF, and NF-κB p65 in the dorsal skin of mice were measured by RT-qPCR. Briefly, the total RNA was separated from dorsal skin by using Trizol regent and reverse transcribed into complementary DNA (cDNA). RT-qPCR was performed on Bio-Rad CFX96 Real-Time System (California, USA) by using the SYBR Green PCR Master Mix kit. The primers used in the animal experiment are listed in Table S2. The mRNA expression values of genes in each sample were normalized to the GAPDH and calculated by using the delta delta threshold cycle (2^−ΔΔCt^) method [48].

#### 4.3.5. Protein Extraction and Western Blot

The dorsal skin was homogenized in RIPA lysis buffer containing 1 mM PMSF. After centrifugation, the supernatant was collected and the total protein concentration was determined by the Lowry method [49]. Proteins with equal amounts were separated by 10% gels for sodium dodecyl sulfate polyacrylamide gel electrophoresis (SDS-PAGE) and then transferred into PVDF membranes. After being blocked with 5% BSA for 2 h at room temperature, the PVDF membranes were probed with the indicated primary antibody overnight at 4 °C, and then incubated with horseradish peroxidase conjugated secondary antibody at room temperature for 1 h. The protein bands were visualized by using an ECL kit and scanned by using the Tanon 5200 MultiImage System (Tanon, Shanghai, China). The densities of the bands were quantified by using Image J software (v1.53i, National Institutes of Health, Bethesda, MD, USA).

### 4.4. In Vitro Cell Culture Experiments

#### 4.4.1. Cell Culture and Treatment

HaCaT cells were purchased from China Center for Type Culture Collection (Wuhan, China) and RAW264.7 was kindly provided by Professor Wu Yuzhou (Hubei Key Laboratory of Bioinorganic Chemistry and Materia Medica, School of Chemistry and Chemical Engineering, Huazhong University of Science and Technology, Wuhan 430074, China). Both cell lines were cultured in DMEM supplemented with 10% FBS, 100 U/mL penicillin, and 100 µg/mL streptomycin in a humidified incubator containing 5% CO_2_ at 37 °C. LPS (1 μg/mL) was used to induce inflammation in HaCaT and RAW264.7 cells.

#### 4.4.2. Cell Viability Assay

MTT assay was used to measure the cell viability with slight modification [50] Briefly, after treatment with SeP or LPS for indicated time, the cells were incubated with 0.5 mg/mL MTT at 37 °C for 4 h. Then, the medium was carefully removed, and the formed formazan products were dissolved with dimethyl sulfoxide (DMSO). The absorbance at 570 nm was measured using an Infinite M200 PRO microplate reader (Tecan, Switzerland).

#### 4.4.3. RT-qPCR and Western Blot Assay

After treatment with LPS and SeP for the indicated time, the total RNA in cells were extracted and the mRNA levels of IL-17A, IL-6, and TNF-α in cells were determined by RT-qPCR as described above. The primers used in the cell culture experiment are listed in Table S2. The mRNA expression value in each sample was normalized to the β-actin gene expression value.

After treatment with LPS and SeP for the indicated time, the total proteins in cells were extracted on ice using the RIPA lysis buffer containing PMSF. After being centrifugated at 12,000 rpm for 15 min at 4 °C, the supernatants were collected for Western blot analysis as described above.

### 4.5. Statistical Analysis

The experimental results from at least three independent experiments were presented as the mean ± standard deviation (mean ± SD) and processed by using SPSS 25.0. One-way analysis of variance (ANOVA) was used to analyze differences among groups. A value of *p* < 0.05 was considered significant.

## 5. Conclusions

This finding demonstrated that SeP had a protective effect against psoriasis. Mechanically, SeP inhibited the expression levels of key inflammatory mediators via the blocking of MAPK and NF-κB signaling pathways, resulting in the exacerbation of IMQ-induced psoriasis-like skin inflammation. Overall, although more detailed investigations are still awaited to understand the pharmacokinetics and pharmacodynamics of SeP, it may be a novel potential therapeutic approach to psoriasis.

## Data Availability

Not applicable.

## Supplementary Materials

The following supporting information can be downloaded at: https://www.mdpi.com/article/10.3390/ijms23042112/s1.
