# Supplementary material for: Selenium-Rich Yeast Peptide Fraction Ameliorates Imiquimod-Induced Psoriasis-like Dermatitis in Mice by Inhibiting Inflammation via MAPK and NF-κB Signaling Pathways
Source: Int J Mol Sci. 2022 Feb 14;23(4):2112. doi: 10.3390/ijms23042112 (PMC8875820; doi:10.3390/ijms23042112)
Supplement: Supplementary file 1 [file ijms-23-02112-s001.zip › ijms-1565167-supplementary.pdf]

Supplemental material

Selenium-rich yeast peptide fraction ameliorates imiquimod-induced psoriasis-like dermatitis in mice by inhibiting MAPK and NF-κB signaling pathways

Hengke Guo[1], Min Li[1], Hongmei Liu[1,2,] *

[1] Hubei Key Laboratory of Bioinorganic Chemistry and Materia Medica, School of Chemistry and Chemical Engineering, Huazhong University of Science and Technology, Wuhan 430074, People's Republic of China

[2] Hubei Engineering Research Center for Biomaterials and Medical Protective Materials, Wuhan, 430074, China

* Corresponding author. Prof. Hongmei Liu, School of Chemistry and Chemical Engineering, Huazhong University of Science and Technology, Wuhan 430074, China

E-mail: hongmeiliuhust@hust.edu.cn; Tel: +86 27 87543032; Fax: +86 27 87543632

Table S1 Sequences and relative area of some peptides in SeP

Peptide sequence	Length	MW（Da）	Relative area
VLPVPF	6	670.41	3369900000
LLPF	4	488.30	3312100000
FFPM	4	540.24	2484800000
LTPTFV	6	676.38	2381100000
ALDDPFLA	8	860.43	2157600000
DLLPV	5	555.33	2125900000
VELLL	5	585.37	2029400000
LLPW	4	527.31	1767400000
LLDLL	5	585.37	1735400000
GVPGDFNLA	9	888.43	1654600000
LGVMLVG	7	687.40	1639100000
DSLVMP	6	660.32	1593400000
PDDFVLS	7	791.37	1548600000
LGDLPF	6	660.35	1541200000
VTPFVPL	7	771.45	1488800000
LDADGLL	7	716.38	1460000000
LDLL	4	472.29	1430000000
VFLPV	5	573.35	1390000000
VGPGDFDLA	9	889.42	1240000000
VIDSTGVFT	9	938.48	1230000000
ALDDPFLA	8	860.43	1210000000
LALPL	5	525.35	1190000000

Note: Relative area refers to the relative integrated area of the peptide content obtained by LC-MS/MS detection, indicating the relative abundance of the peptide in SeP.

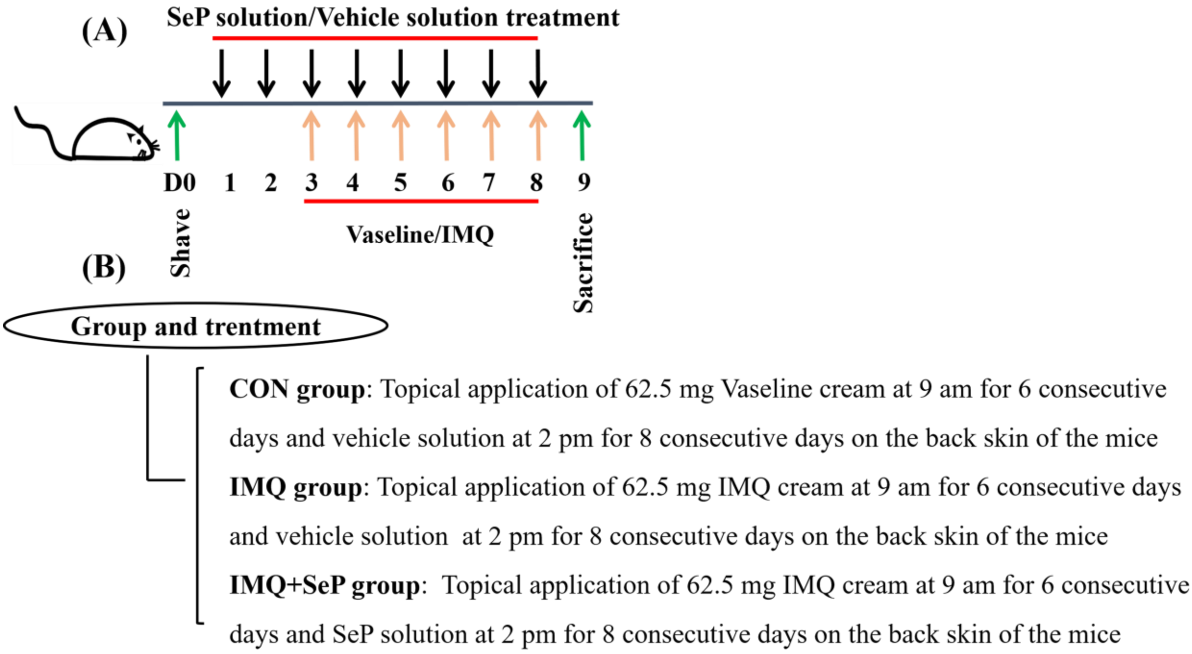

(B)

Group and trentment

CON group: Topical application of 62.5 mg Vaseline cream at 9 am for 6 consecutive days and vehicle solution at 2 pm for 8 consecutive days on the back skin of the mice

IMQ group: Topical application of 62.5 mg IMQ cream at 9 am for 6 consecutive days and vehicle solution at 2 pm for 8 consecutive days on the back skin of the mice

IMQ+SeP group: Topical application of 62.5 mg IMQ cream at 9 am for 6 consecutive days and SeP solution at 2 pm for 8 consecutive days on the back skin of the mice

Scheme S1. The schematic design of animal experiments

Table S2 Primers sequences used for the detection of related gene expression

	Gene	Sense Primer (5’ to 3’)	Anti-sense Primer (5’ to 3’)
Mouse	TNF-α	ACC CTC ACA CTC AGA TCA TCT T	GGT TGT CTT TGA GAT CCA TGC
	IL-17A	TTT AAC TCC CTT GGC GCA AAA	CTT TCC CTC CGC ATT GAC AC
	IL-17F	TGC TAC TGT TGA TGT TGG GAC	AAT GCC CTG GTT TTG GTT GAA
	IFN-γ	GTT ACT GCC ACG GCA CAG TCA TTG	ACC ATC CTT TTG CCA GTT CCT CCA G
	VEGF	GCA CAT AGG AGA GAT GAG CTT CC	CTC CGC TCT GAA CAA GGC T
	IL-23	TCC TCC AGC CAG AGG ATC ACC C	AGA GTT GCT GCT CCG TGG GC
	CXCL-1	TAG GGT GAG GAC ATG TGT GG	AAA TGT CCA AGG GAA GCG T
	P65	GTT TCG GGT AGG CAC AGC AAT	TCG AGT CTC CAT GCA GCT ACG
	IL-1β	CAC TAC AGG CTC CGA GAT GAA	TGT CGT TGC TTG GTT CTC CT
	GAPDH	GGG CTC TCT GCT CCT CCC TGT	CGG CCA AAT CCG TTC ACA CCG
Human	TNF-α	ATG TTG TAG CAA ACC CTC AAG C	CCT TGA AGA GGA CCT GGG AGT A
	IL-17A	GCC TTC AAG ACT GAA CAC CG	TGA CAT GCC ATT CCT CAG GG
	IL-23	CTC CCT GAT AGC CCT GTG	GAC TGA GGC TTG GAA TCT
	IL-6	CTG AGG GCT CTT CGG CAA AT	GCC CAG TGG ACA GGT TTC TG
	β-actin	CCT GGG CAT GGA GTC CTG TG	TCT TCA TTG TGC TGG GTG CC
